# Elements of General Pathology

**Published:** 1843-04

**Authors:** 


### Art. VI.

*Elements of General Pathology*. By the late John Fletcher, m.d. Edited by John J. Drysdale, m.d., and John R. Russell, m.d.—*Edinburgh*, 1842. 8vo, pp. 519.

That a sound and readable treatise on general pathology, sufficiently elevated in its character to be of value as a scientific work, and yet not too much raised above the comprehension of the average student-mind of the present generation, is one of the greatest desiderata in our medical literature, few will be disposed to deny. How far we consider that the volume before us supplies the deficiency, will presently appear. We must, in the first place, very much question the judgment of the editors in submitting to the world, after an interval of six years, during which the aspect of pathological science has undergone many important alterations, a treatise which it may be safely affirmed that its author himself would not have published in its present form. The materials put into their hands were nothing more than "notes of lectures, often scarcely intelligible;" and rather than take any such liberties with these, as it seems to us that the necessary transformation of them would have fully warranted, "they have scrupulously adhered to the author's text, and have added in editorial notes what seemed necessary to completeness, and sufficient to put the reader in possession of what has been done in the science up to the present time." Now if it had been of any great consequence to the world to know the exact opinions of Dr. Fletcher on any given subject, if he had possessed the profoundly-philosophic intellect of a Haller, or the all-grasping intuition of a Hunter, this would certainly have been the proper course. But neither in physiology nor in pathology could we regard with satisfaction, as a proper guide to the learner, the writings of *any* man, however eminent in his time, which had remained during a period of at least six years of this eventful epoch, unmodified save by editorial additions. Such additions, if faithfully made, must in many instances directly contradict the text;* and we need not say how perplexing to the student this kind of instruction must be.

* See Dr. Bostock's Physiology, 3d edition, for many instances of this kind. No person can have a higher value than ourselves for this work, as a learned collection of the opinions of the most eminent physiologists; but few, we think, would now regard it as anything but a sort of bibliographical record.

The perusal of the volume before us has not in any respect altered the opinion of the character of Dr. Fletcher's mind, which we formerly expressed when reviewing his Rudiments of Physiology. (vol. V. pp. 93-8.) We find in it examples of the same closeness of reasoning, the same ingenuity of argument, the same acuteness in exposition; but we also find instances of the same superficial dogmatism, the same carelessness as to the data on which the reasoning is founded, and the same overweening confidence in the superior value of his own conclusions. Did we think that the work was likely to become a popular one, we should feel it necessary to point out these, for the sake of putting our readers on their guard against its specious fallacies. But as we think that its influence will be too limited to render this necessary, we shall satisfy ourselves with a concise analytical view of its contents, stopping to notice briefly whatever we deem most worthy of remark for its excellence or the opposite.

The work is divided into three books, devoted to Etiology, Semeiology, and Therapeutics, respectively. It seems to us that many of the subjects included under the first two of these heads have no claim to their position. Thus, after the *remote causes*—the *exciting causes*—the influence of the various vital stimuli, as *light*, *electricity*, *air*, *aliment*—the mental stimuli, as *sympathy* and the *passions*—accidental stimuli, as *hemorrhage*, *venoms*, &c., and the *excretions*, *exercise*, and *sleep*, have been discussed, the author carries us on through the proximate cause, (which, if we regard disease as a *morbid action*, and not as a collection of symptoms, is nothing else than the disease itself,) to *inflammation*, with its many theories—to *fever*, no less prolific, thence to *increased secretion—natural tissues in excess—tubercle* and *cancer—calculi* and *deposition of fluids—increased depositions of halitus and blood—preternatural fluids*—and lastly *functional proximate causes*, among which are included all the spasmodic actions. It seems to us, and probably will to our readers, that in the introduction of all the latter group of subjects under etiology, there is an extraordinary jumble of ideas, which we can scarcely attribute to so clear a mind as Dr. Fletcher's. That it may have proceeded from the editors we are led to apprehend, by finding several important novelties regarding the introduction of organic agents into the body, (such as parasitic growths, ferments, &c.) inserted in the chapter on light, electricity, and air, instead of in that on accidental stimuli, in which they would have been grouped along with the animal poisons, to which they have the clearest analogy. We are surprised also to find appended to this same chapter, an account of Andral's late researches on the alterations which the blood undergoes in disease, although that subject is clearly akin to all those which occupy the later portions of the book.

Similar inconsistencies are encountered when we go into detail. Thus in p. 2, note, the author incidentally tells us that "neither health (i. e. natural action) nor disease (i. e. morbid action) are entities, but abstractly signify different modes of being, or the same mode of being under different circumstances. Whilst in p. 5 we are formally told that "it is surely better to call by the name of a disease such a collection of symptoms; and by the name of a proximate cause such a morbid condition of the body as immediately gives rise to them." We do not think it of much consequence which definition is adopted; but whichever it be, it ought to be strictly adhered to. There is a certain practical convenience in applying the term disease to the collection of symptoms; but in a scientific treatise on pathology, we apprehend that it is scarcely possible to maintain this application throughout, except with a most undesirable amount of circumlocution; and we find our author in many places departing from his formal definition, and going back to his incidental one.

Of the degree in which this work gives a correct view of the present aspect of the science of which it treats, we may adduce a very characteristic example from chapter ii. where we find the following denunciation of the humoral pathology:

"Others have endeavoured, at least partially, to renew the effete theory that these causes operate by producing some primary change in the blood; but it is unnecessary to dwell particularly on the merits of a system so totally vague.... Of the existence of any primary change in the properties of blood, as resulting from the exciting causes of disease, there is neither proof nor probability. Besides, *it is not the blood, but the solids, that are the seat of disease;* so that the blood, even if primarily affected, can be regarded only as the last link in the chain of exciting causes, and these causes must still operate upon the solids before they can produce their effect." (p. 48.)

This dogmatic assertion, the fallacy of which is now so palpable, is in harmony with our author's equally dogmatic assertion in his physiology, that the blood is totally destitute of vitality; a doctrine in which we apprehend that he would no longer find any support from those who are acquainted with modern science. It must be well known to our readers that the tendency of nearly all pathological researches during the last few years has been to reestablish the humoral pathology on a sounder basis, and with a more accurate knowledge of the actual changes involved; and that, as if to prove that "there is nothing new under the sun," the very terms *fermentation* and *putrefaction* of the fluids, on which so mnch ridicule has been cast, are now found most accurately to indicate the nature of the morbid alterations which they undergo. Of the facility with which Dr. Fletcher frames hypotheses upon the loosest data, whilst exposing the inconsistencies of others (in which respect he strongly resembles Magendie), the following quotation may serve as an example:

"It is remarkable that the protoxide of nitrogen, previously to occasioning asphyxia, commonly gives rise to a kind of delirium, attended with very pleasing sensations, and an uncontrollable propensity to laughter. The cause of this is, unquestionably, a specific irritation excited in the lungs by this peculiar stimulus, which is conveyed to the brain in the manner of sympathy; in the same way as ordinary intoxication is to be ascribed to a specific irritation excited in the stomach by spirituous liquors, and conveyed to the brain in a similar manner." (p. 64.)

The editors have not here added a note to contradict their text, as they have frequently done elsewhere; but they certainly might have known, from one of the most important prize theses which their university has produced, that the influence of alcohol on the functions of the brain is not due to a "specific irritation conveyed to the brain in the manner of sympathy;" but, as Dr. Percy has given strong reason for believing, to the direct action of the alcohol on the nervous matter. Our author has subsequently carried out the same erroneous view, in regard to the action of all poisons. Thus he remarks that

"The fact cannot be too forcibly insisted on, that we have no evidence whatever, that any primary change in the blood (the presumed necessity of which has been principally instrumental in propagating the vague notion respecting the absorption of poisons,) is at all essential to the action of any one of them. It is much more probable, that when poisons operate on parts at a distance from the organ to which they have been directly applied, they do so by exciting in the organ a peculiar irritation, which, when conveyed by sympathy to the other parts of the body, produces its principal effects on that part which is most adapted by its specific irritability to be so acted upon." (p. 101.)

On this head the editors have endeavoured to do justice to the reader by adding a long note, in which many of Dr. Fletcher's objections to the doctrine of absorption of poisons are answered; but we will simply ask, Is this the proper mode of teaching pathology? In a subsequent chapter the same doctrines are inculcated by Dr. Fletcher, in regard to the non-absorption of the poisons of syphilis, variola, hydrophobia, &c., which he believes to act as specific stimuli only on the parts to which they are first applied, and sympathetically as to all those at a distance. We need not tell our readers how many important facts have been accumulated during the last few years, which prove the erroneous nature of this doctrine; and we much regret that they are not here noticed by the editors, who content themselves with referring to the previous discussion on the operation of poisons in general.

It is rather amusing to contrast Dr. Fletcher's own explanations with those which, when given by others, he so acutely ridicules. Thus when speaking of the opinions of Dr. Ewings that dyspepsia "consists primarily of muscular spasm, membranous irritation, and nervous uneasiness," he remarks that "it unluckily displays too much observational sinisterity, ratiocinatorial impotence, and phraseological unintelligibility to be made anything of, and is one of the best examples of the substitution of words for ideas, even in the fruitful field of pathology," (p. 393, note.) Again, when alluding to the doctrine of a recent nosologist, who refers subsultus tendinum to the "principle of irritation, which is often apt to accumulate in the tendinous extremities of the muscles," he remarks: "A man would leave a warehouse with a particularly clear idea of the mechanism and powers of the crane employed in raising bales of goods, if told by the person employed to instruct him, that the said bales were raised by a principle of irritation, often apt to accumulate in the ropes attached to them; yet of a precisely similar nature is the vague and unmeaning trash which is often held out by pretended medical theorists under the head of explanation," (p. 443, note.) Does not this just severity, we would ask, apply equally well to our author's own attempt at explaining the action of morbific agents, as a "local specific irritation, conveyed in the manner of sympathy to distant parts?"

We have left ourselves but brief space to enumerate the contents of the second book. The first chapter, on nosology, has for its purpose chiefly to prove that systematic nosology is a useless science; in which we quite agree with him, so long as definitions of disease are to be founded upon symptoms and not upon the nature of the morbid action they involve. The heads of the succeeding chapters are as follows: *Anatomical symptoms—respiration—auscultation of respiration—circulating system—digestion—sensations—thought—voluntary motion.* The whole of the portion referring to auscultation has been rewritten by the editors, who are well-known followers of the doctrines of Skoda; and altogether this book seems to us to be much less faulty than the preceding, and to contain much that the student will not find elsewhere brought together in the same intelligible form.

The last book, on Therapeutics, may be considered disproportionately short, as it only occupies about one seventh of the whole volume; and the numerous subjects of which it treats are certainly disposed of in a very summary manner. The author commences by pointing out that therapeutics is strictly a branch of pathology; since the agents of which it treats have the same kind of bearing on morbid conditions of the body, that heat, air, aliment, &c. have upon the body in health, a doctrine which we took occasion to bring forwards some years since. (vol. VI. p. 109.) He then considers the general indications for treatment, and especially prophylactic treatment; under which head the editors introduce a short account of the German "Wasser-cur." The second chapter treats of the general action of medicines, and is liable to the same exceptions which we have already taken against our author's doctrine of poisons and morbific agents. At its close are some acute observations on the homœopathic system, which seem to us to analyse the strange mixture of truth and error it contains in a very judicious manner. The editors give, in a long note, a fuller account of the history and progress of the doctrine; and intimate that "having paid considerable attention to the question of the applicability of the homœopathic principle to practice, they have arrived at conclusions somewhat different from, and more favorable than, those deduced from theoretical grounds alone by Dr. Fletcher." (p. 498.) In the third and fourth chapters, our author takes a general survey of the different classes of medicines; but this is much too brief to be either practically or scientifically useful to the student. He treats with much ridicule the attempts which are being made to reduce medicinal agents to their simplest forms; and justly regrets that "any compound medicine of acknowledged efficacy should ever be wantonly expunged and voted inert, because we cannot explain why it should be otherwise." But he says nothing of the many advantages which attend this simplification, both as regards their scientific study of the modus operandi of the various agents upon the system, and with respect to their application in practice. In this, as in other instances, it must be remembered that "ridicule is not the test of truth," even though it should appear so apposite as that contained in the following sentence, with which the volume concludes, and with which we shall close our quotations from it:

"It is the same lopping and pruning spirit, aided by the recent improvement in the art of chemical analysis, which has led to the rejection in many cases, not only of compounds in general, but even of crude simples, in favour of one or two of their proximate principles, in which we are pleased to consider that all their virtues reside; and there can be little doubt that, if their improvement proceed much farther, a middle-sized man will not only be able to take all the medicine he may have occasion for on the point of a needle, but, instead of sitting down, as at present, to his pound-and-a-half of mutton chops and pot of porter, will swallow for his dinner a fine [five?] grain pill, composed of equal parts of ovine and cerevisine, or some such matters, which science has yet to be delivered of."

It may be thought that, in cur account of this volume, we have too far transgressed the laudable principle "de mortuis nil nisi *bonum*." But we much prefer the version of it which we were accustomed to learn in our youth from the lips of one of the most upright and benevolent of men, "de mortuis nil nisi *verum*." We have felt called upon to express a decided opinion as to the character of this work, and the probability of its injurious influence upon those who may peruse it without the advantage of a previous more correct knowledge of the subject. Painful as this opinion may be to the friends of Dr. Fletcher, we cannot but lament that they have drawn it upon themselves by a publication which, we feel assured, *he* would never have permitted to go forth in its present form, and which *they* would have best consulted his reputation by leaving undisturbed. We have felt it necessary to expose some of his errors, because we are confident that so far as his opinions are received they must operate to the retardation rather than the advance of science. And we have adverted to defects in the character of Dr. Fletcher as a philosopher, because we would present him, to our younger readers more especially, as an example of the fact, that no closeness of reasoning can be relied on, so long as the data are unsound; that no acuteness in perceiving and exposing error can be of any avail, as long as it is exercised only towards others and not against self; and that no deductions can be sound, in which any facts that bear upon them are excluded from consideration. That many valuable facts and many important suggestions are brought together in this treatise, no one who duly appreciates Dr. Fletcher's character can doubt; and we may add that much of the matter interpolated by the editors is well deserving of attention, being derived from sources which are but little known in this country.

---

